# How Does the Involuntary Choice of Self-Employment Affect Subjective Well-Being in Small-Sized Business Workers? A Cross-Sectional Study from the Fifth Korean Working Conditions Survey

**DOI:** 10.3390/ijerph19021011

**Published:** 2022-01-17

**Authors:** SangJin Park, Chulyong Park, Joo Hyun Sung

**Affiliations:** 1 Department of Occupational and Environmental Medicine, Gyeongsang National University Changwon Hospital, College of Medicine, Gyeongsang National University, Jinju 52727, Korea; oemdrsjpark@gnuh.co.kr; 2 Department of Occupational and Environmental Medicine, Yeungnam University Hospital, Daegu 42415, Korea; ironyong@yu.ac.kr; 3 Department of Preventive Medicine and Public Health, College of Medicine, Yeungnam University, Daegu 42415, Korea; 4 Department of Occupational and Environmental Medicine, Institute of Health Sciences, Gyeongsang National University Changwon Hospital, College of Medicine, Gyeongsang National University, Jinju 52727, Korea

**Keywords:** self-employment, involuntary retirement, subjective well-being, mental health, depression, Korean Working Conditions Survey

## Abstract

In South Korea, self-employed workers comprise 24.6% of the working population—among which 99.7% were found to operate with less than 50 employees. However, few studies have investigated the effects of an involuntary choice of self-employment. In this study, based on the fifth Korean Working Conditions Survey, the factors affecting subjective well-being and mental health in small business owners with less than 50 employees among service/sales workers, who account for more than half of the self-employed population in Korea, were analyzed by the World Health Organization—Five Well-Being Index, using the Student’s *t*-test, ANOVA and logistic regression analysis. Results showed that the well-being level for those who opted for self-employment involuntarily was lower than those who chose it voluntarily. Then, participants were separated into two groups according to sex; the degree corresponding to the poor well-being score group was compared, and that of the group who chose self-employment because they could not find any other work was significantly higher than that of the group who chose it voluntarily, in both men and women, and this was similar even after correcting for covariance. As the number of people being forced to choose self-employment is expected to further increase after the outbreak of COVID-19, future studies should be conducted to improve subjective well-being of such workers.

## 1. Introduction

In South Korea, self-employed workers comprise a high proportion of the working population. With self-employed workers representing 24.6% of the national workforce in 2019, Korea ranks eighth among the 37 member countries of the Organization for Economic Co-operation and Development (OECD). This rate is four times higher than that of 6.1% in the United States—the lowest percentage among OECD countries—and 1.5 times higher than the average of 15.2% from 27 countries in the European Union [1]. Among the self-employed in Korea, small business owners with 1–4 employees accounted for the highest percentage of 70.2%, followed by 23.9% with 5–9 employees, 5.2% with 10–29 employees and 0.4% with 30–49 employees [2]. Therefore, 99.7% of all self-employed workers were found to operate with less than 50 employees. In addition, the proportion of self-employed persons increased by 76,987 compared to the previous year 2018, an increase of 6.3%. Among them, while the number of businesses with 1–4 and 5–9 employees increased by 7.1% and 6.3%, respectively, businesses with 10–29 and 30–49 employees decreased by 3.6% and 1.5%, respectively [2]. As such, most self-employed businesses in South Korea operate on a small size, and the proportion of small businesses is gradually increasing. This may be the effect of the gig economy (the tendency of companies to hire independent contractors and freelancers instead of full-time employees), which is emerging globally in response to rapidly changing times. In fact, according to the 2018/2019 global report of the Global Entrepreneurship Monitor (GEM), the proportion of Korean adults participating in these activities was 21.5%—the highest among the 54 countries surveyed [3].

In South Korea, voluntary and involuntary retirements, such as honorary retirement and layoffs, are increasing because of prolonged economic instability and a worsening trade economy. While most companies stipulate that the retirement age is 60–65 years, as of May 2021, the age at which workers quit their longest-serving job was 51.2 years for men and 47.7 years for women [4]. Since most of those who retire at this age leave work before they are eligible to receive pensions (e.g., in the case of the National Pension, those born after 1969 can receive their pension from the age of 65), they are exposed to the risk of economic poverty until they receive a pension [5,6]. Therefore, to avoid this risk, there might be workers who involuntarily enter self-employment.

There have been a few studies which have shown that self-employed workers suffer less emotional stress than employed workers [7,8]. It is known that this is because self-employed workers have a higher degree of discretion and freedom of work compared to employed workers. However, in South Korea, unlike in other countries, the proportion of small-sized self-employed workers is significantly higher, and a few studies have shown that self-employed workers suffer from more mental health problems [9,10,11,12]. One of the causes for this may be the situation in which one has to involuntarily choose self-employment, as mentioned above. The involuntary choice of self-employment can be a serious mental and psychological burden on workers, and can be expected to have a significant impact on their subjective well-being and mental health. However, research on the effect of such situations on workers’ well-being and mental health is insufficient. In particular, the situation of self-employed workers is getting worse due to the prolonged worldwide economic instability following the COVID-19 outbreak [13,14]; in fact, in the first half of 2020, when the World Health Organization (WHO) declared COVID-19 to be a global pandemic, the number of sole proprietors in Korea increased by 26.8% compared to the previous year [15]. Therefore, there is a need for further research on autonomy for the choice of self-employment affecting the well-being and mental health of self-employed workers in South Korea.

In this study, based on the World Health Organization—Five Well-Being Index (WHO-5), we investigated the factors that affect the well-being and mental health of service/sales workers, who account for more than half of the self-employed population [2], especially those who involuntarily choose self-employment, and what measures are needed to improve them.

## 2. Materials and Methods

### 2.1. Study Population

This study used data from the fifth Korean Working Conditions Survey (KWCS). The fifth KWCS was conducted at the Korea Occupational Safety and Health Research Institute (OSHRI) during approximately 18.5 weeks from 11 July to 17 November 2017. The KWCS was developed based on the European Working Conditions Survey (EWCS) and the Labour Force Survey (LFS) in the United Kingdom [16,17]. The survey has been conducted five times, from 2006 to 2017, and the sixth survey is currently underway. The validity and reliability of the KWCS have been verified in previous studies [18,19]. The survey was conducted by professional researchers who had completed interview training. One-on-one interviews were conducted with members of selected households through household visits by professional researchers. As a survey tool, the Blaise program, a statistical survey system for computer-assisted personal interview (CAPI), developed by Statistics Netherlands, was introduced, and an electronic questionnaire mounted on a tablet PC was used instead of the existing paper survey table. Data were collected using the paper and pen interview (PAPI) method only when it was impossible to proceed due to a malfunction of the tablet PC. In total, 50,205 people from 17 areas responded to the survey [17].

Among the self-employed people, service/sales workers who worked at only one site in the private sector were selected; furthermore, 9139 among them were selected from small-sized businesses with less than 50 employees. Depending on the reason for choosing to start their own business, respondents who answered “Totally through own preference” and those who said “Couldn’t find any other work” were selected. Additionally, 6141 workers between their 30s and 50s who were actively engaged in economic activities were selected. Finally, 6022 workers (2270 men, 3752 women) were included in the analysis for this study, excluding those with missing values for education level, monthly income, working hours per week and the WHO-5 well-being index.

### 2.2. Survey

In this study, the following data were used from the fifth KWCS to identify the general characteristics of the participants: gender, age, education level, occupation, monthly income, number of employees, working hours per week and the reason for starting their own business. We analyzed the level of subjective well-being and poor well-being score group such as the score 13 and below among the participants using the items from the WHO-5 well-being index, which were included in the questionnaire.

#### 2.2.1. General Characteristics

We stratified variables to identify the level of well-being and the poor well-being score group of the participants as follows. The participants were selected from the economically active group, and ages were classified as “30–39”, “40–49” and “50–59” years. As regards the education level of the subjects, only 2.9% had middle school graduation or less. Therefore, we divided them into “high school graduate or below”, and “college or above”. Monthly income was stratified into “under 2000”, “2000–2990”, “3000–3990” and “4000 or above” in thousands of Korean Republic won (KRW 1000 = USD 0.86, as of September 2021). Occupation was limited to service and sales jobs. Service workers included caregivers, hairdressers, wedding planners, fortune tellers, chefs and cooks, sales workers, included car salespeople, insurance solicitors, store cashiers, sales promoters, Internet salespeople, food salespeople and telemarketers. In the case of the number of employees, more than half of the subjects had been one-person workers (58.0%); 2–4 had accounted for 36.0%; whilst others accounted for 6.0%. Therefore, the number of employees was stratified into “1 person only” “2–4 people” and “5–49 people”. Working hours per week were divided into groups working for 52 h or less and working for more than 52 h, based on the legal limit of 40 working hours per week, plus 12 h, which is an additional allowable overtime, under the Korean Labor Standards Act [20].

The following question was used to determine the subjective well-being level and poor well-being score group according to the reason for choosing self-employment: “Have you chosen to be self-employed due to your own preference, or because you could not find any other work?” The responses were: (1) totally through own preference; (2) could not find any other work; (3) a combination of both; (4) neither of these reasons; (8) do not know (spontaneous); and (9) refusal (spontaneous). Of these, only responses of “1” and “2” were selected to compare the differences between the two groups.

#### 2.2.2. WHO-5 Well-Being Index

The fifth KWCS questionnaire contained five questions related to the WHO-5 well-being index. The participants were instructed as follows: “From the following questions, choose the one that best describes how you have been feeling over the past two weeks: (A) I have felt cheery and in good spirits, (B) I have felt composed and comfortable, (C) I have felt energetic and lively, (D) I have woken up refreshingly in the morning because my fatigue was gone and (E) My daily life has been full of interesting things to me”. The raw score was calculated as the sum of the answers: (1) always; (2) most of the time; (3) more than half of the two weeks; (4) less than half of the two weeks; (5) sometimes; and (6) at no time. The raw scores ranged from 5 to 30. We then converted the raw score into the WHO-5 well-being index score. A score of 0 represented the worst possible quality of life and a score of 25 represented the best possible quality of life. A score of 13 or below not only implied poor well-being, but was also an indication recommending testing for depression under the International Classification of Diseases (ICD)-10.

### 2.3. Statistical Analysis

We used the Student’s *t*-test to analyze the WHO-5 well-being level according to the participants’ sex, education level, working hours per week and reason for choosing self-employment. A one-way analysis of variance (ANOVA) followed by a Scheffe multiple comparison test for post hoc comparisons was used to compare subjective well-being levels for age, monthly income and the number of employees. In addition, the participants were divided according to sex, logistic regression analysis was used to identify the poor well-being score group for each variable, and multivariate logistic regression analysis was used to correct the influence of covariance on the reason for choosing self-employment, such as age, education level, monthly income, working hours per week and the number of employees.

We used IBM SPSS Statistics for Windows, version 24.0 (IBM, SPSS Inc., Armonk, NY, USA) to conduct the Student’s *t*-test, the ANOVA test, the logistic regression analysis, and the multivariate logistic regression analysis; a *p*-value lower than 0.05 was considered statistically significant.

## 3. Results

### 3.1. Comparison of Subjective Well-Being Level

In total, data from 3407 participants (1878 men and 1529 women) were analyzed after adjusting for weight. The results showed that there was no significant difference in subjective well-being between men and women. The age of the subjects was 49.18 ± 7.99 (*p* < 0.001). When compared according to age, it was found that subjective well-being level was significantly better for participants in their 30s than for those in their 50s. According to the education level, participants who graduated college or above showed significantly higher well-being scores than those who only graduated high school or below; participants with a monthly income of KRW 4,000,000 or more showed a significantly higher level of well-being than others. There were no significant differences in the level of well-being based on the number of employees. The working hours per week of the subjects were 55.57 ± 14.59 (*p* < 0.001), which was longer than the legal working hours of wage workers. When compared according to working hours per week, the well-being score of those who worked 52 h or less was significantly higher than those who worked for more than 52 h. Compared to those who voluntarily chose self-employment, those who could not find any other work showed a significantly lower level of well-being (Table 1).

### 3.2. Poor Well-Being Score Group

According to the statistics of the Ministry of Health and Welfare in 2016, the lifetime prevalence of depression among Koreans differs significantly by sex (3.0% male, 6.9% female) [21]; therefore, we separated the participants by sex in this analysis. In addition, the poor well-being group, those with a score of less than 13 points on the WHO-5 well-being index, was analyzed. In the case of men, it was found that the well-being of those in their 50s was significantly higher than that of those in their 30s (odds ratio (OR): 1.35, 95% confidence interval (CI): 1.01–1.83). Furthermore, the higher the education level of the men, the lower it was (OR: 0.56, 95% CI: 0.45–0.69). For the group with a monthly income of KRW 4,000,000 or more, it was found to be significantly less than that for the group with an income of under KRW 2,000,000 (OR: 0.39, 95% CI: 0.26–0.60). It was found that those who chose self-employment because they could not find any other job were more than twice as many as those who voluntarily chose to be self-employed (OR: 2.02, 95% CI: 1.37–2.97). In the case of women, similar results were found: it was also found to be significantly higher in their 50s than in their 30s (OR: 1.57, 95% CI: 1.13–2.17), and the higher the education level, the lower it was (OR: 0.64, 95% CI: 0.50–0.81). This was found to be significantly lower for the groups with a monthly income of KRW 3,000,000–3,990,000 and more than KRW 4,000,000 than the group with a monthly income of less than KRW 2,000,000, and among the three groups, it was observed that the higher the income, the lower the odds ratios (OR: 0.71, 95% CI: 0.51–0.99; OR: 0.54, 95% CI: 0.37–0.79). In the case of women, unlike men, the odds ratio was found to be higher for the group working more than 52 h per week than for those working 52 h or less per week (OR: 1.43, 95% CI: 1.13–1.81). The participants belonging to the poor well-being score group were at least 2-fold more depending on the reason for choosing self-employment (OR: 2.23, 95% CI: 1.37–3.63) (Table 2).

In order to analyze the participants belonging to the poor well-being score group according to the reason for self-employment selection, in this study, each variable was selectively corrected and analyzed again. First, when adjusted for age, education level, monthly income and working hours per week, which were found to have a significant correlation in the above analysis (Model 2), in both men and women, the odds ratio for those who chose self-employment because they could not find any other work was found to be significantly higher than for those who voluntarily chose it (men aOR (adjusted odds ratio): 1.74, 95% CI: 1.16–2.59; women aOR: 2.10, 95% CI: 1.28–3.46). Similar results were also obtained when the number of employees was added and corrected (Model 3) (men aOR: 1.74, 95% CI: 1.16–2.59; women aOR: 2.11, 95% CI: 1.28–3.47). In men, in both models, a higher education level indicated a significantly and proportionately lower proportion of participants belonging to the poor well-being score group (Model 2 aOR: 0.62, 95% CI: 0.49–0.78; Model 3 aOR: 0.62, 95% CI: 0.49–0.77). In addition, in both men and women, the monthly income groups earning KRW 4,000,000 or more were found to be lower than the group earning under KRW 2,000,000 in Model 2, (men aOR: 0.48, 95% CI: 0.31–0.73; women aOR: 0.56, 95% CI: 0.38–0.83) and similar results were found in Model 3 (men aOR: 0.47, 95% CI: 0.30–0.72; women aOR: 0.54, 95% CI: 0.36–0.81). In women, the group who worked more than 52 h was higher than the other group in both models (Model 2 aOR: 1.42, 95% CI: 1.11–1.81; Model 3 aOR: 1.40, 95% CI: 1.09–1.79) (Table 3).

## 4. Discussion

The purpose of this study was to investigate the effect of the reason for choosing self-employment on the subjective well-being and mental health of self-employed service/sales workers. The results showed that the group who chose self-employment because they could not find any other work showed a significantly lower level of well-being than the group who voluntarily chose to be self-employed. The odds ratios of the poor well-being score group for the group who chose self-employment because they could not find any other work were found to be at least 2-fold that of the group who voluntarily chose self-employment, in both men and women before the correction, and were also found to be 1.74 times higher for men and 2.10 for women after the correction. In addition, it was found that the odds ratios of poor well-being score groups with a monthly income of more than KRW 4,000,000 were only approximately half of those with an income of less than KRW 2,000,000; 0.48 times for men and 0.56 times for women. In women, when the weekly working hours exceeded 52 h, the ratio was found to be 1.40 times or more.

In general, several studies have shown that self-employed workers are less emotionally stressed than other workers because they have greater work discretion and self-efficacy [7,8]. However, as mentioned in the introduction section, due to the characteristics of Korea, which has a high proportion of small business owners, it has been found that self-employed workers have more mental health problems than workers employed elsewhere [9,10,11,12]. In this study, 25.83% (880/3, 407) of the participants belonged to the poor well-being score group, which was more than a quarter of all small-sized self-employed workers. It is estimated that such a high percentage is influenced by having to opt for self-employment against one’s will, the stress from self-employment operations, cultural factors and socio-economic conditions.

In self-employed workers with small businesses, in many cases, employers have to personally conduct various tasks related to administration and business management. In the cases of choosing self-employment entirely based on one’s own preference, it can be assumed that the psychological burden of administrative work and business management will be relatively small. However, for workers who were forced to choose self-employment because they could not find any other job, such tasks could be a huge psychological burden. In fact, several studies have shown that in workplaces with fewer employees, as the workloads given to individuals increase, the responsibilities of work also increase, resulting in higher work-related stress [22,23,24]. However, if the business thrives and income increases, it would become possible to hire employees to help with various tasks, which can reduce the burden on employers; in this case, subjective well-being and mental health can also be expected to improve.

One peculiarity of the results of this study was that in women, unlike men, working hours per week had a significant effect on subjective well-being. This result is presumed to be due to the fact that the burden of housework is greater for women than for men in Korea [25]. The burden of housework is further increased due to such long working hours; as a result, it can be expected to adversely affect their mental health. Moreover, as mentioned above, the lifetime prevalence of depression in women is higher than that in men [21], which might have also affected the results. Although it is not possible to generalize these results, it is expected that a similar pattern would be shown in countries where the burden of housework is greater on women, as in Korea.

The situations in which another job could not be found may be the result of a decrease in trade volume due to interest rate fluctuations or the spread of infectious diseases such as COVID-19, and the resulting economic downturn. According to the World Trade Organization (WTO), logistics movement decreased immediately after the outbreak of COVID-19 in 2020, resulting in a worsening trade economy [26]. As a country with a heavy dependence on exports, South Korea responds more sensitively to such international situations (2019 export dependence rate of 32.9% in South Korea, 7.7% in the USA and 13.9% in Japan; export dependence is the total export/GDP) [27]. As mentioned in the introduction, in Korea, the number of sole proprietors increased by 26.8% compared to the previous year in the first half of 2020, when the WHO declared the COVID-19 to be a pandemic [15]. Therefore, there is a possibility that the subjective well-being and mental health of small-sized self-employed workers may be worse than those found in this study. The survey period of the fifth KWCS was 2017, which was not affected by the pandemic conditions. Therefore, further studies should be conducted based on these results to identify the factors that may affect the subjective well-being and mental health of those who have chosen self-employment against their will.

This study had several limitations. First, since this was a cross-sectional study, it was not possible to compare the differences in the level of well-being and mental health status of workers before and after choosing self-employment; thus, although it was found that the involuntary choice of self-employment is associated with poor well-being and mental health, the results cannot prove a causal relationship. Since the study was targeted at small business owners with less than 50 employees, it was not possible to analyze the self-employed workers with more than 50 employees, for which health managers exist under the Occupational Safety and Health Act in South Korea [28]. In addition, since it was difficult to ascertain individual medical health conditions of the subjects, such as hypertension and diabetes, the effects of these problems on subjective well-being and mental health could not be considered. Additionally, although the questionnaire included workplace psychological well-being such as job satisfaction and work stress, it was difficult to quantify because most of the contents of fifth KWCS were related to wage workers. Moreover, since the participants were limited to service and sales workers, the results cannot be applied to self-employed workers in other industries. However, the results of this study are significant because the participants’ well-being was analyzed using the WHO-5 well-being index, which is a widely used measure. In addition, according to our investigation, many studies have been conducted on the factors affecting the mental health of self-employed workers in Korea, but few studies have investigated the effects of an involuntary choice of self-employment. The subjective well-being and mental health of small-sized self-employed workers can be improved through further research on the conditions after the COVID-19 outbreak. Furthermore, such research can be helpful in making related policy decisions.

## 5. Conclusions

It was found that the subjective well-being of small-sized workers who chose self-employment against their will was significantly lower than that of the workers who voluntarily chose self-employment, and was significantly more likely to be an indication for the depression test. Involuntary self-employment could increase in the economic downturn due to several situations, such as the COVID-19 pandemic; therefore, through future policies, support for self-employed workers should be carefully considered, with accompanying actions to improve their subjective well-being and mental health.

## Figures and Tables

**Table 1:** General characteristics of the study populations and their subjective well-being score (*N* = 3407).

Variables	Subjective Well-Being Score			*p*-Value	Post Hoc Comparison
	Number	Mean	SD		
Sex				0.400	–
Male	1878	15.0	4.8		
Female	1529	14.8	4.9		
Age				<0.001	c < a
30–39 ^a^	618	15.4	4.7		
40–49 ^b^	1203	15.1	4.9		
50–59 ^c^	1586	14.6	4.9		
Education level				<0.001	–
High school graduation or below	1925	14.4	5.0		
College graduation or above	1482	15.6	4.7		
Monthly Income (in thousands of KRW)				<0.001	d, e, f < g
<2000 ^d^	411	14.2	5.0		
2000–2990 ^e^	865	14.6	4.9		
3000–3990 ^f^	1004	14.7	5.1		
≥4000 ^g^	1127	15.6	4.6		
Number of employees				0.149	–
1 only	1975	14.8	5.0		
2–4	1229	15.0	4.7		
5–49	203	15.3	5.0		
Working hours per week				0.001	–
52 h or less	1316	15.3	4.7		
More than 52 h	2091	14.7	5.0		
Reason for choosing self-employment				<0.001	–
Totally through own preferences	3221	15.0	4.8		
Couldn’t find any other work	187	13.2	5.2		

SD: standard deviation; Student’s *t*-test was used to analyze for sex, education level, working hours per week and reason for choosing self-employment; ANOVA was used to analyze for age, monthly income and the number of employees; Post hoc comparison based on Scheffe multiple comparison test; KRW 1,000,000 ≒ USD 840 ≒ EUR 730; The superscript letters within the table are for Post Hoc comparison.

**Table 2:** Odds ratios for the poor well-being score groups according to associated factors.

Variables	Male			Female		
	Number	OR	(95% CI)	Number	OR	(95% CI)
Age						
30–39	340	1.00		278	1.00	
40–49	663	1.17	(0.86–1.60)	540	1.08	(0.77–1.53)
50–59	875	1.35	(1.01–1.83)	711	1.57	(1.13–2.17)
Education level						
High school graduation or below	947	1.00		978	1.00	
College graduation or above	931	0.56	(0.45–0.69)	551	0.64	(0.50–0.81)
Monthly income (in thousands of KRW)						
<2000	115	1.00		295	1.00	
2000–2990	312	0.71	(0.45–1.12)	553	0.81	(0.60–1.10)
3000–3990	608	0.73	(0.48–1.11)	397	0.71	(0.51–0.99)
≥4000	843	0.39	(0.26–0.60)	284	0.54	(0.37–0.79)
Number of employees						
1 only	934	1.00		1041	1.00	
2–4	803	0.83	(0.67–1.04)	426	0.96	(0.74–1.23)
5–49	141	0.91	(0.60–1.37)	62	0.58	(0.30–1.12)
Working hours per week						
52 h or less	721	1.00		595	1.00	
More than 52 h	1157	1.20	(0.97–1.50)	934	1.43	(1.13–1.81)
Reason for choosing self-employmwnt						
Totally through own preference	1761	1.00		1460	1.00	
Couldn’t find any other work	117	2.02	(1.37–2.97)	69	2.23	(1.37–3.63)

OR: odds ratio; CI: confidence interval; KRW 1,000,000 ≒ USD 840 ≒ EUR 730.

**Table 3:** Adjusted odds ratios for the poor well-being score groups after adjusting for covariance.

Variables	Model 1 ^a^		Model 2 ^b^		Model 3 ^c^	
	Male	Female	Male	Female	Male	Female
	OR (95% CI)	OR (95% CI)	aOR (95% CI)	aOR (95% CI)	aOR (95% CI)	aOR (95% CI)
Reason for choosing self-employment						
Totally through own preference	1.00	1.00	1.00	1.00	1.00	1.00
Couldn’t find any other work	2.02 (1.37–2.97)	2.23 (1.37–3.63)	1.74 (1.16–2.59)	2.10 (1.28–3.46)	1.74 (1.16–2.59)	2.11 (1.28–3.47)
Age						
30–39			1.00	1.00	1.00	1.00
40–49			1.09 (0.79–1.51)	1.01 (0.70–1.44)	1.09 (0.79–1.50)	1.00 (0.70–1.43)
50–59			1.09 (0.80–1.50)	1.32 (0.93–1.89)	1.09 (0.79–1.49)	1.32 (0.92–1.89)
Education level						
High school graduation or below			1.00	1.00	1.00	1.00
College graduation or above			0.62 (0.49–0.78)	0.78 (0.59–1.03)	0.62 (0.49–0.77)	0.77 (0.59–1.02)
Monthly income (in thousands of KRW)						
<2000			1.00	1.00	1.00	1.00
2000–2990			0.74 (0.46–1.17)	0.83 (0.61–1.14)	0.74 (0.47–1.17)	0.82 (0.60–1.12)
3000–3990			0.84 (0.55–1.29)	0.75 (0.53–1.06)	0.85 (0.55–1.31)	0.72 (0.51–1.02)
≥4000			0.48 (0.31–0.73)	0.56 (0.31–0.73)	0.47 (0.30–0.72)	0.54 (0.36–0.81)
Working hours per week						
52 h or less			1.00	1.00	1.00	1.00
More than 52 h			1.11 (0.89–1.39)	1.42 (1.11–1.81)	1.13 (0.90–1.42)	1.40 (1.09–1.79)
Number of employees						
1 only					1.00	1.00
2–4					1.00 (0.79–1.25)	1.16 (0.88–1.53)
5–49					1.35 (0.87–2.07)	0.76 (0.38–1.51)

OR: odds ratio; aOR: adjusted odds ratio; CI: confidence interval; KRW 1,000,000≒ USD 840 ≒ EUR 730; ^a^ Model 1: unadjusted; ^b^ Model 2: adjusted for age, education level, monthly income and working hours per week; ^c^ Model 3: adjusted for age, education level, monthly income, working hours per week and number of employees.

## Data Availability

Raw data of KWCS are available from the following URLs: https://oshri.kosha.or.kr/eoshri/resources/KWCSDownload.do (accessed on 6 January 2022).
